# Possible Antiviral Activity of 5-Aminolevulinic Acid in Feline Infectious Peritonitis Virus (Feline Coronavirus) Infection

**DOI:** 10.3389/fvets.2021.647189

**Published:** 2021-02-10

**Authors:** Tomomi Takano, Kumi Satoh, Tomoyoshi Doki

**Affiliations:** Laboratory of Veterinary Infectious Disease, Department of Veterinary Medicine, Kitasato University, Towada, Japan

**Keywords:** FIP, coronavirus, 5-aminolevulinic acid, antiviral drug, cat

## Abstract

Feline infectious peritonitis (FIP) is a life-threatening infectious disease of cats caused by virulent feline coronavirus (FIP virus: FIPV). For the treatment of FIP, several effective antivirals were recently reported, but many of these are not available for practical use. 5-amino levulinic acid (5-ALA) is a low-molecular-weight amino acid synthesized in plant and animal cells. 5-ALA can be synthesized in a large amount, and it is widely applied in the medical and agricultural fields. We hypothesized that 5-ALA inhibits FIPV infection. Therefore, we evaluated its antiviral activity against FIPV in felis catus whole fetus-4 cells and feline primary macrophages. FIPV infection was significantly inhibited by 250 μM 5-ALA. Our study suggested that 5-ALA is applicable for the treatment and prevention of FIPV infection.

## Introduction

Feline infectious peritonitis (FIP) is a life-threatening infectious disease caused by feline coronavirus (FCoV) in domestic and wild Felidae species. FCoV is highly prevalent worldwide in cats. FCoV is an enveloped, single strand positive-sense RNA virus. This virus belongs to the genus *Alphacoronavirus* in the subfamily *Orthocoronavirinae* of the family *Coronaviridae* (1). FCoV is divided into two serotypes based on the amino acid sequence of the spike (S) protein, serotype I FCoV, and serotype II FCoV (2). Serological and genetic surveys revealed that type I FCoV is dominant worldwide (3–5). FCoV is mainly spread by fecal-oral transmission (6). Most FCoV-infected cats are subclinical. However, several mutations occurred in the S protein, leading to development of the virulent type called feline infectious peritonitis virus (FIPV) (7, 8). The hallmark pathological findings of FIP in cats are serous fluid in peritoneal and pleural cavities, and pyogranulomatous lesions in several organs (9).

FIP is an immune-mediated and difficult-to-treat virus infection. Several effective antivirals for FIP treatment were recently reported (10, 11), but many are not available for practical use. Some anti-FCoV drugs, such as itraconazole, are available at animal hospitals, but their treatment effects are limited (12). As FIP is a chronic and systemic disease, it is difficult to achieve clinical remission. Accordingly, it is desirable that therapeutic drugs for FIP have the following characteristics: (1) Few side-effects for cats, (2) low price, and (3) low mutagenesis of pathogens.

5-amino levulinic acid (5-ALA) is a low-molecular-weight amino acid synthesized in plant and animal cells (13, 14). It is an intermediate in biosynthesis of tetrapyrrole. As 5-ALA is highly water-soluble and low cytotoxic, it is widely applied in the medical and agricultural fields (15). Several studies on the effects of 5-ALA on infectious disease have been reported. Suzuki et al. reported that when 5-ALA and ferrous ion were orally administered to rodent malaria parasite (*Plasmodium yoelii*)-infected mice, the mice survived (16). On the other hand, its effects on viral infection are unclear.

In veterinary medicine, photodynamic therapy (PDT) using 5-ALA has been investigated for tumor treatment in dogs (17), but to our knowledge, the effects of 5-ALA on infectious diseases in animals have not been investigated. We investigated whether 5-ALA can be applied as an anti-FCoV drug *in vitro*.

## Materials and Methods

### Cell Cultures, Animals, and Viruses

*Felis catus* whole fetus (fcwf)-4 cells (kindly supplied by Dr. M. C. Horzinek of Universiteit Utrecht) were grown in Eagles' MEM containing 50% Leibovitz's L-15 medium, 5% fetal calf serum (FCS), 100 U/ml of penicillin, and 100 μg /ml of streptomycin. The maintenance medium was the same composition as the growth medium except for the concentration of FCS (2%). For primary macrophages, feline primary macrophages were selected. Feline alveolar macrophages were obtained from four specific-pathogen-free (SPF) cats aged 3–5 years by bronchoalveolar lavage with Hank's balanced salt solution. Feline primary macrophages were maintained in D-MEM supplemented with 10% FCS, 100 U/mL of penicillin, and 100 μg/mL of streptomycin. SPF cats were maintained in a temperature-controlled isolated facility. The experiment using animals was approved by the President of Kitasato University through judgment of the Institutional Animal Care and Use Committee of Kitasato University (18-050) and performed in accordance with the Guidelines for Animal Experiments of Kitasato University. Sample sizes were determined based on our previous study and the minimum number of cats was used. The type I FCoV KU-2 strain (FIPV-I KU-2) was isolated in our laboratory. FCoV-II 79-1146 was kindly provided by Dr. M. C. Horzinek of Utrecht University. These viruses were grown in fcwf-4 cells at 37°C.

### Compounds

5-ALA and sodium ferrous citrate (SFC) were obtained from Neopharma Japan (Tokyo, Japan). 5-ALA and SFC were dissolved in maintenance medium at 200 and 50 mM, respectively. SFC solution was used as a solvent of 5 ALA. On the day of the experiments, these compounds were diluted to the desired concentrations in maintenance medium.

### Cytotoxic Effects of Compounds

The fcwf-4 cells were seeded on 96-well plates. The compounds were added in triplicate to the wells. After incubation for 96 h, the culture supernatants were removed, WST-8 solution (Kishida Chemical, Osaka, Japan) was added, and the cells were returned to the incubator for 1 h. The absorbance of formazan produced was measured at 450 nm using a 96-well spectrophotometric plate reader, as described by the manufacturer. Percentage cell viability was calculated using the following formula: Cell viability (%) = [(OD of compound-untreated cells - compound-treated cells)/ (OD of compound-untreated cells)] × 100. The 50% cytotoxicity concentration (CC_50_) was defined as the cytotoxic concentration of each compound that reduced the absorbance of treated cells to 50% when compared with that of the untreated cells.

### Antiviral Effects of 5-ALA

Confluent fcwf-4 cell monolayers were cultured in medium with or without compounds at the indicated concentrations in 24-well multi-plates at 37°C for 24 or 48 h. Cells were washed and the virus (MOI 0.01) was adsorbed into the cells at 37°C for 1 h. After washing, cells were cultured in 1.5% carboxymethyl cellulose (CMC)-MEM or MEM with or without compounds. In the case of cells cultured in CMC-MEM, the cell monolayers were incubated at 37°C for 48 h, fixed, and stained with 1% crystal violet solution containing 10% buffered formalin, and the resulting plaques were then counted. The percentage of the decrease or increase in plaques was calculated using the following formula: Percentage of the plaque reduction (%) = [(plaque number of compound-treated cells) / (plaque number of compound-untreated cells)] × 100. The EC_50_ was defined as the effective concentration of compounds that reduced the virus titer in the culture supernatant of infected cells to 50% when compared with that of the virus control. In the case of cells cultured in MEM, the culture supernatants were collected 48 h post-infection and virus titers were measured by the TCID_50_ assay.

Primary feline macrophages were cultured in medium with or without compounds at the indicated concentrations in 24-well multi-plates at 37°C for 48 h. After washing with PBS, FIPV 79-1146 (1 × 10^4^ TCID_50_) was allowed to adsorb to the cells at 37°C with 5% CO_2_ for 1 h. After washing with PBS, the cells were cultured in the medium and the supernatants were collected. The virus titers were measured by the TCID_50_ assay.

### Statistical Analysis

Data from only two groups were analyzed using the Student's *t*-test (Welch's *t*-test) and those of multiple groups were analyzed by one-way ANOVA followed by Tukey's test. A *P*-value of < 0.05 was considered significant.

## Results

### Cytotoxic and Antiviral Effects of 5-ALA

**Figure 1:**
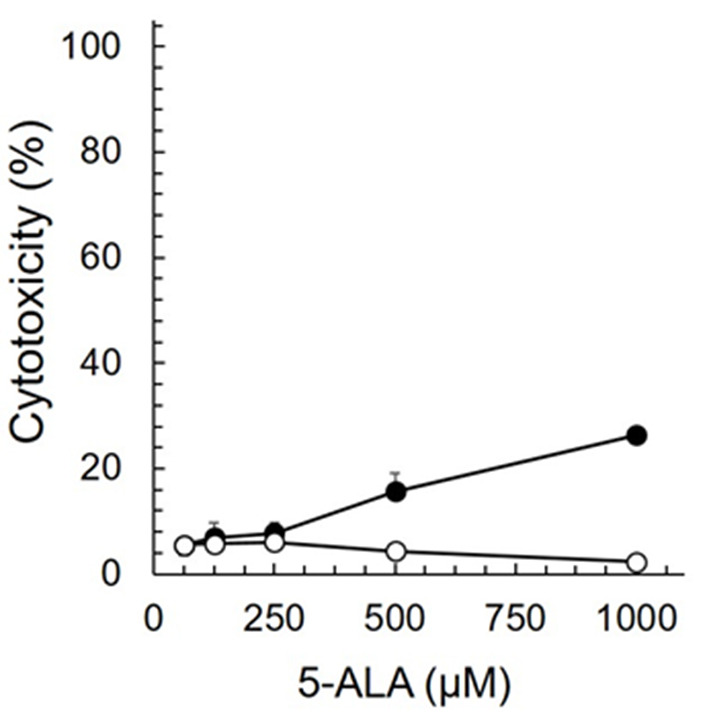
Cytotoxic effects of 5-ALA in fcwf-4 cells. Fcwf-4 cell viability was measured by WST-8 assay. Black circle: 5-ALA. White circle: Vehicle (SFC). The vehicle (solvent control) was the same as that in 5-ALA solution at each serial dilution. The results are shown as the mean ± SE. Data represent three independent experiments (*n* = 3).

Cytotoxicity assay was performed to clarify the non-toxic concentration of 5-ALA against fcwf-4 cells (Figure 1). More than 75% of fcwf-4 cells survived in the presence of 1,000 μM 5-ALA (the maximum concentration in this experiment). Vehicle control exhibit no cytotoxic effects on fcwf-4 cells.

### The Effects of 5-ALA on FIPV Infection in Feline Cell-Line

**Figure 2:**
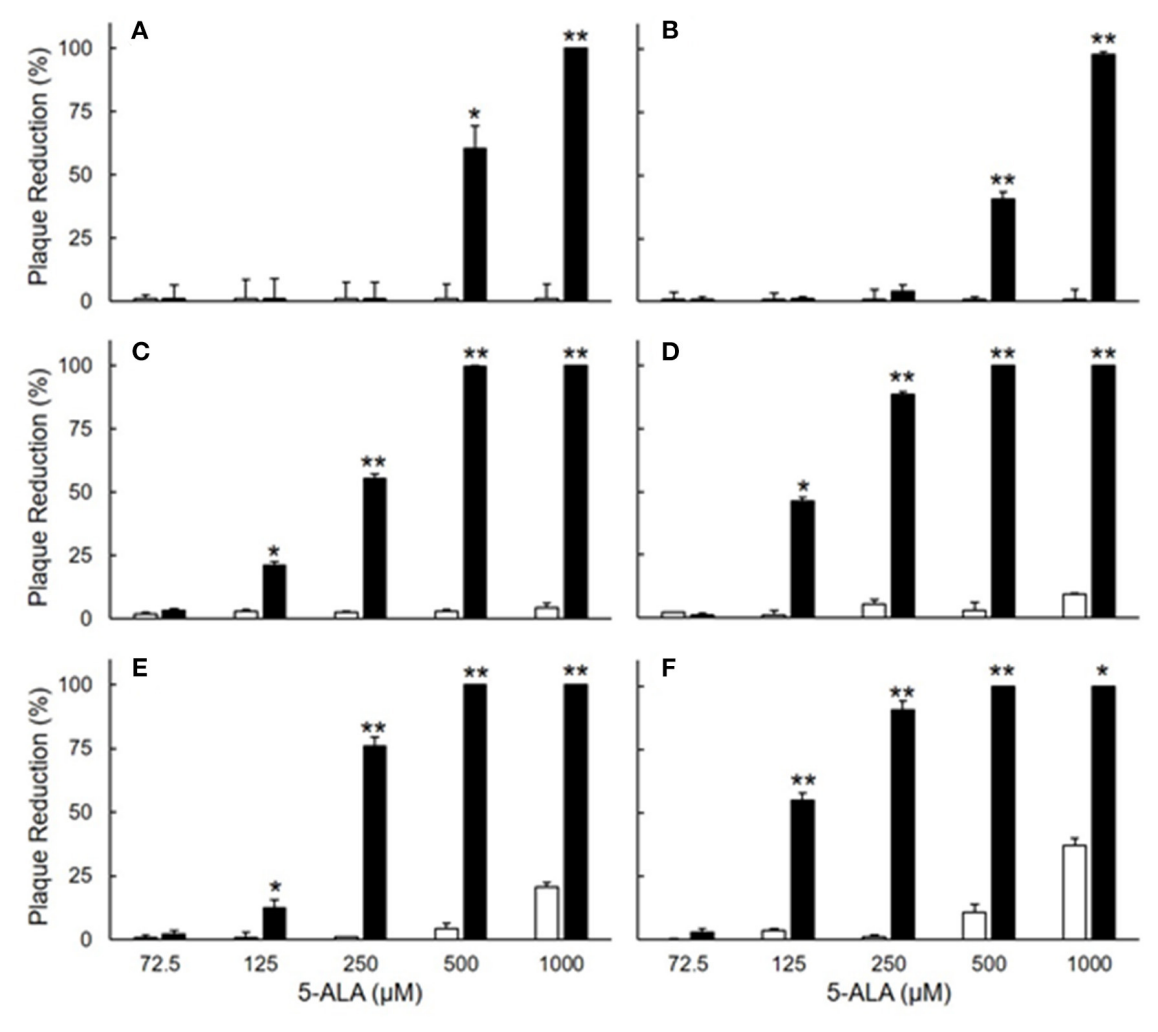
Plaque inhibition assay of FIPV in fcwf-4 cells treated with 5-ALA. **(A,B)** Effects of 24-h pre-treatment on antiviral activity of 5-ALA. The rate of plaque inhibition of FIPV-infected fcwf-4 cells pre-treated with 5-ALA for 24 h. **(C,D)** Effects of 24-h pre-treatment and 48-h post-treatment on antiviral activity of 5-ALA. The rate of plaque inhibition of FIPV-infected fcwf-4 cells pre-treated for 24 h and post-treated for 48 h with 5-ALA. **(E,F)** Effects of 48-h pre-treatment and 48-h post-treatment on antiviral activity of 5-ALA. The rate of plaque inhibition of FIPV-infected fcwf-4 cells pre-treated for 48 h and post-treated for 48 h with 5-ALA. **(A,C,E)** type I FIPV. **(B,D,F)** type II FIPV. Black bar: 5-ALA. White bar: Vehicle (solvent control). The results are shown as the mean ± SE. Data represent four independent experiments (*n* = 4). **p < 0.01 (*p < 0.05) vs. vehicle.

**Figure 3:**
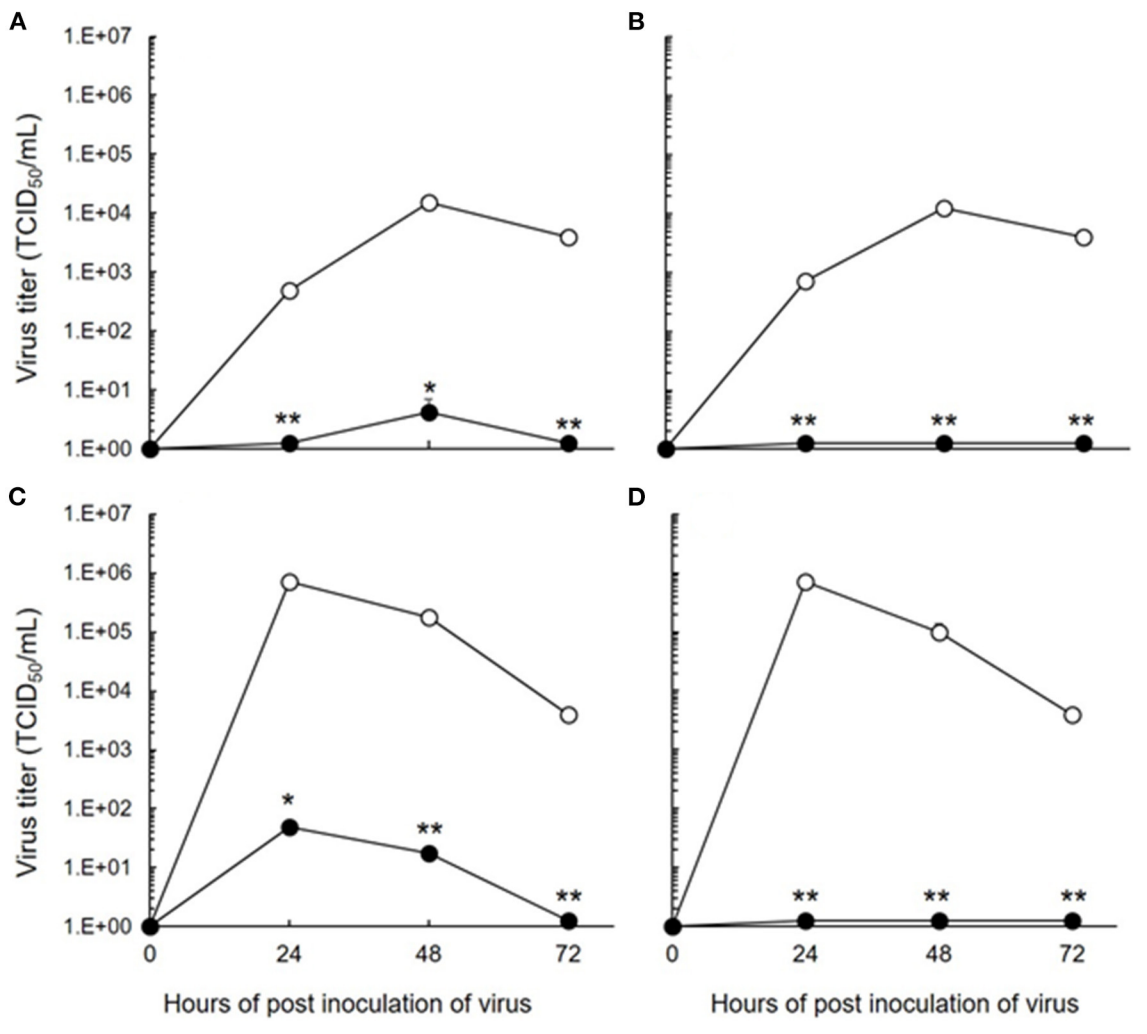
Inhibition of FIPV infection by 5-ALA in fcwf-4 cells. **(A,C)** Effects of 48-h pre-treatment and 48-h post-treatment on antiviral activity of 250 μM 5-ALA. **(B,D)** Effects of 48-h pre-treatment and 48-h post-treatment on antiviral activity of 500 μM 5-ALA. **(A,B)** 250 μM 5-ALA. **(A,B)** type I FIPV. **(C,D)** type II FIPV. Black circle: 5-ALA. White circle: Vehicle (solvent control). The results are shown as the mean ± SE. Data represent four independent experiments (*n* = 4). **p < 0.01 (*p < 0.05) vs. vehicle.

The antiviral effects of 5-ALA against FIPV were evaluated by plaque inhibition assay in fcwf-4 cells. Cells were treated with 5-ALA through the following 3 procedures: 24-h pre-treatment (pre-24 h), 24-h pre-treatment followed by 49-h co-treatment with FIPV (pre-24 h and co-49 h), and 48-h pre-treatment followed by 49-h co-treatment with FIPV (pre-48 h and co-49 h). In pre-24 h, the percentage of plaque inhibition significantly increased at 500 μM or higher (Figures 2A,B). In pre-24 h and co-49 h, the percentage of plaque inhibition significantly increased at 125 μM or higher (Figures 2C,D). In pre-48 h and co-49 h, the percentage of plaque inhibition in type I FIPV by 125 μM 5-ALA reached 75% (Figures 2E,F). Vehicle control, SFC, exhibited no plaque-inhibitory effects on FCoV under any condition. According to the titration assay, the production of type I and type II FIPV was significantly reduced by 250 and 500 μM 5-ALA (Figure 3).

### The Effects of 5-ALA on FIPV Infection in a Feline Cell Line

**Figure 4:**
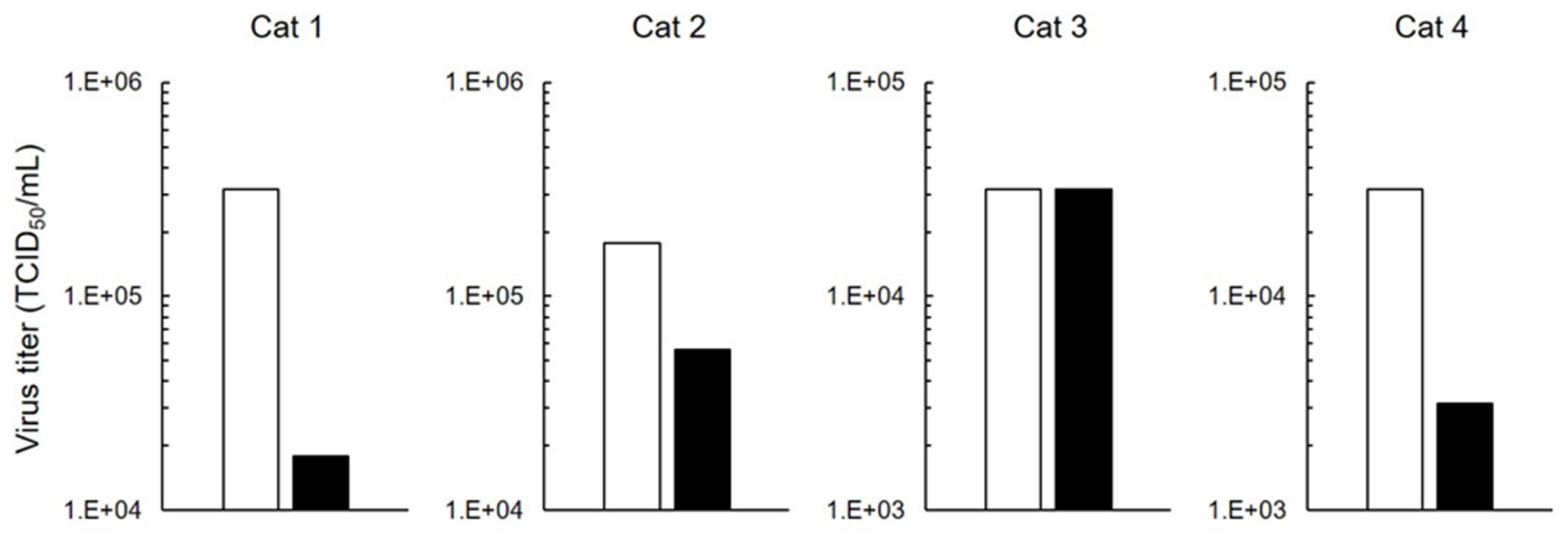
Inhibition of FIPV infection in macrophages. Effects on antiviral activity of 5-ALA (250 μM) in feline primary macrophages. Black bar: 5-ALA. White bar: Vehicle (solvent control). Data represent two independent experiments (*n* = 3).

FIPV-infected macrophages are involved in the progression of FIP symptoms to a severe state. We investigated whether 5-ALA inhibits FIPV multiplication in macrophages. In this experiment, type II FIPV 79-1146 with high ability of multiplication in feline primary macrophages was used. Virus production in FIPV-infected macrophages was reduced by 250 μM 5-ALA in three of four cats (Figure 4).

## Discussion

5-ALA is an intermediate of tetrapyrrole synthesis in animals, plants, and microorganisms (13–15). In the 1980's, the possibility of efficacy of 5-ALA in plants was reported (18), but it was difficult to produce 5-ALA in a sufficient amount for practical use because only a small amount is produced in microorganisms. After Nishikawa et al. established a mass production method of 5-ALA using bacteria (19), the effectiveness of 5-ALA was confirmed in not only agriculture, but also in medical and biological fields. 5-ALA is inexpensive, and it is practically used as a supplement to improve animal performance and immune response in the field of veterinary medicine (14, 20).

5-ALA inhibited the growth of FIPV in fcwf-4 cells. Metal complexes of a 5-ALA metabolite, protoporphyrin IX (PpIX), have been reported to have anti-virus activity (21–23). A PpIX metal complex, heme, inhibits dengue virus multiplication (21). On the other hand, multiplication of Zika virus is not inhibited by heme (23). It is unclear whether heme inhibits FCoV multiplication. Generally, an increase in intracellular heme stimulates the production of hemeoxygenase-1 (HO-1), the heme-degrading enzyme. HO-1 has been reported to induce anti-viral activity (24, 25). However, we confirmed in a preliminary experiment that the HO-1 mRNA expression level was unchanged in cells treated with 250 μM 5-ALA (Data not shown). Based on this, 5-ALA-induced inhibition of FIPV infection occurs due to a factor other than heme and HO-1.

There have been many recent studies on therapeutic drugs for FIP. Many drugs effective for FIP *in vitro* were identified and several have been confirmed to exhibit treatment effects when administered to cats with FIP (10–12). However, the effects of all drugs were poor in cases with neurological manifestations. As a reason for this, poor transfer of these drugs to the central nervous system was considered; therefore, a drug exhibiting anti-viral effects against FIPV able to reach brain tissue is needed. 5-ALA is a low-molecular-weight amino acid and can transfer to brain tissue (26). In addition, it has been reported that the diffusion of 5-ALA from blood to normal brain tissue is very low (27), suggesting that it exhibits fewer adverse effects. FIP can be definitely diagnosed only by detection of the FCoV antigen within lesion (28). However, when treatment is initiated after making a definite diagnosis, symptoms have progressed and the condition does not respond to treatment in many cases. Therefore, if a drug that can be prophylactically administered before diagnosing FIP is available, progression of symptoms may be prevented, for which 5-ALA may be ideal agent. However, in our experiment using the target cells of FIPV, macrophages, the antiviral effects of 5-ALA were not observed in some cats. Therefore, when 5-ALA is used as a therapeutic drug for FIP, anti-FCoV drugs, such as GS-441524 (29), GC-376 (30), U18666A (31), and itraconazole (32), or anti-inflammatory drugs, such as anti-TNF-alpha antibody (33), should be concomitantly used.

In the field, FECV is mainly transmitted between cats, whereas horizontal infection of FIPV between cats is considered rare (34). FIPV was suggested to be generated by genetic mutation of FECV. Thus, if there are means to prevent FECV infection on a daily basis, the development of FIP may be prevented. No vaccine capable of preventing FECV infection has been developed. Addie et al. reported that virus gene excretion in feces disappeared in FECV-infected cats treated with a synthetic adenosine analog (35). Therefore, removal of FECV infecting the intestine by antiviral administration to FECV-infected cats is expected. However, synthetic adenosine analogs may induce coronavirus gene mutation (36). Moreover, even though the FECV gene level in feces decreased to below the detection limit in cats treated with a synthetic adenosine analog, it is possible for FECV to latently infect the intestine or other tissues. To prevent FECV gene mutation and reliably eradicate FECV infection, long-term synthetic adenosine analog administration is necessary, but it is not realistic because this drug is too expensive. On the other hand, 5-ALA is practically used as a supplement. 5-ALA has low toxicity in animals and plants, strongly suggesting that long-term administration of 5-ALA to cats is possible. It is necessary to investigate whether 5-ALA is applicable as a supplement to prevent the development of FIP in FECV-infected cats in the future.

In this study, we confirmed the possibility that 5-ALA inhibits FIPV multiplication and TNF-alpha production. As 5-ALA is an amino acid present in the body, its immediate administration is possible. However, it is necessary to administer 5-ALA to cats with FIP and observe whether therapeutic effects can be acquired. Furthermore, whether long-term administration of 5-ALA eliminates the virus and inhibits FIP development in FECV-infected cats must be investigated.

## Data Availability Statement

The original contributions presented in the study are included in the article/supplementary material, further inquiries can be directed to the corresponding author/s.

## Ethics Statement

The animal study was reviewed and approved by the President of Kitasato University through judgment of the Institutional Animal Care and Use Committee of Kitasato University.

## Author Contributions

TT conceived and designed the study, analysed the data, and wrote the manuscript. TT and KS collected the data. TT, KS, and TD collected and processed the samples. All authors read and approved the final manuscript.

## Conflict of Interest

The authors declare that the research was conducted in the absence of any commercial or financial relationships that could be construed as a potential conflict of interest.
